# Somatization Symptoms Regulate Emotional Memory Bias in Adolescents With Major Depressive Disorder

**DOI:** 10.3389/fpsyt.2021.656198

**Published:** 2021-08-26

**Authors:** Mo Daming, Li Xin, Hu Shuwen, Guo Pengfei, Liu Shuai, Geng Feng, Cao Xiaomei, Chen Binbin, Zhong Hui

**Affiliations:** ^1^Department of Psychiatry, Affiliated Psychological Hospital of Anhui Medical University, Hefei, China; ^2^Anhui Mental Health Center, Hefei, China; ^3^Hefei Fourth People's Hospital, Hefei, China

**Keywords:** adolescents, depression, emotional, memory, somatization

## Abstract

**Objective:** Somatization symptoms are commonly comorbid with depression. Furthermore, people with depression and somatization have a negative memory bias. We investigated the differences in emotional memory among adolescent patients with depressive disorders, with and without functional somatization symptoms (FSS).

**Methods:** We recruited 30 adolescents with depression and FSS, 38 adolescents with depression but without FSS, and 38 healthy participants. Emotional memory tasks were conducted to evaluate the emotional memory of the participants in the three groups. The clinical symptoms were evaluated using the Hamilton Depression Rating Scale (HDRS) and the Children's Somatization Inventory (CSI).

**Results:** The valence ratings and recognition accuracy rates for positive and neutral images of adolescent patients were significantly lower than those of the control group (*F* = 12.208, *P* < 0.001; *F* = 6.801, *P* < 0.05; *F* = 14.536, *P* < 0.001; *F* = 6.306, *P* < 0.05, respectively); however, the recognition accuracy rate for negative images of adolescent patients of depression without FSS was significantly lower than that of patients with FSS and control group participants (*F* = 10.316, *P* < 0.001). These differences persisted after controlling for HDRS scores. The within-group analysis revealed that patients of depression with FSS showed significantly higher recognition accuracy rates for negative images than the other types (*F* = 5.446, *P* < 0.05). The recognition accuracy rate for negative images was positively correlated with CSI scores (*r* = 0.352, *P* < 0.05).

**Conclusion:** Therefore, emotional memory impairment exists in adolescent patients of depression and FSS are associated with negative emotional memory retention.

## Introduction

Major depressive disorder (MDD) is a chronic disabling condition characterized by abnormal mood changes. The prevalence of MDD increases significantly during adolescence (1) and in results in a high economic burden on society. In the last 20 years the relationship between depression and cognitive impairment in MDD has gained sufficient attention (2). According to Beck's cognitive model of depression, cognitive biases cause individuals with depression to partially remember, perceive, and pay attention to emotions, in particular, they selectively weaken the processing of positive information (3), which plays an important role in the development and maintenance of depression (4).

The relationship between emotion and memory, especially the effect of emotion on memory, has always been the focus of cognitive psychology and neuroscience. Emotional memory is influenced by situational factors that affect the encoding, storage, and retrieval of emotionally stimulating information. Emotional events are easy to remember and have long-lasting and stronger effects than non-emotional (neutral) events, which suggests that emotions can regulate memory efficiency (5). Emotional memory bias can be divided into two types: bias toward negative stimuli and away from positive stimuli (6). Emotional state affects memory function healthy individuals as well as individuals with emotional disorders, who are often impaired by negative emotion (7). Specifically, both adults and adolescents with depression preferentially remember negative information (8–10) and are more likely to have negative emotional memory (11, 12). In a study that utilized an intentional memory task, patients with MDD remembered more negative words than positive words, while the control group showed the opposite results (13).

In addition to understanding the impact of MDD on negative emotional memory, this study also aims to understand functional somatization symptoms (FSS) in individuals with MDD. This study also aims to examine functional somatization symptoms (FSS) in individuals with depression cannot be fully explained by organic pathology (14, 15). FSS are also known as medically unexplained symptoms (MUS), which have been observed in about 25% of children and adolescents in Western countries (16). In China, the incidence of FSS in children was as high as 7.6% (17). Studies in Western countries have found that the incidence of somatization symptoms or symptom groups, in individuals with depression is about 66–93% (18–20). Additionally, a survey of individuals with depression in many East Asian countries, including China, found that somatization symptoms are a common clinical complaint among such patients and more than half of them displaying somatization symptoms (21). Similarly, among children diagnosed with depression, the reported rate of somatization symptoms was twice as high as among those in the control group (22). It should be noted that depressive disorder is a risk factor for somatization symptoms and is not the cause of FSS (23).

An increasing number of studies have reported that the interaction of biological, psychological, and social factors is a common cause of FSS (24–26). Studies of patients with somatoform disorders have shown that their neurocognitive functions of memory, executive function, and attention are impaired (27–30). In addition, studies of children with chronic pain disorders have found that pain symptoms are related to attention and working memory (31). Similarly, patients with somatization disorders have shown preferences for negative memory (32), implying that these patients are more likely to remember somatic symptom words than neutral words. These results suggest that patients with somatization disorders may have both attention and memory bias toward somatic symptom words (33).

In clinical work, we found that many adolescent patients have somatization symptoms, especially adolescents suffering from mood disorders. However, many teenagers go to general hospitals repeatedly due to somatization symptoms. After repeated physical examinations and the advice of general practitioners, they will go to psychiatry or psychological consultation. Some parents do not think somatization symptoms are psychological problems. Therefore, it is of great clinical significance to explore the relationship between depression and somatization symptoms in adolescents. In summary, patients with depression display a memory bias toward negative stimuli or away from positive stimuli. Furthermore, depressive disorders often lead to somatization symptoms, which have the same memory bias toward negative stimuli. However, the memory function in patients of depression with somatization symptoms, especially in adolescent patients, has not been studied. Therefore, we hypothesized that the emotional memory of adolescent patients with depression will differ based on the presence or absence of FSS.

## Methods

### Participants

Two groups of participants were recruited. The patient group consisted of 68 adolescent patients with depression recruited from the psychological hospital affiliated with the Anhui Medical University (Hefei Fourth People's Hospital). Two professional psychiatrists interviewed the participants using the Chinese version of the Structured Clinical Interview for the DSM-IV, and reached a consensus about the participants' diagnoses using all the available information. Inclusion criteria for participants were: (1) conformance with the DSM-IV diagnostic criteria for MDD and (2) Han Chinese ethnicity and aged 13–18 years. In contrast, the exclusion criteria for participants were: (1) past or present diagnosis of another major psychiatric Axis I or Axis II disorder such as schizophrenia, or bipolar affective disorder, etc., (2) consumption of any psychotropic drugs within 4 weeks, or history of alcohol or substance abuse, (3) history of substantial physical illness such as head trauma, neurological illness, pain, cardiovascular disease or Gastrointestinal diseases, depression patients have completed clinical examinations such as blood routine, biochemistry, thyroid function, electrocardiogram, brain topography, abdominal color Doppler ultrasound, head CT or MRI at the time of enrollment. (4) medical conditions that could lead to psychiatric symptoms, (5) a standard score of <24 on Raven's Standard Progressive Matrices, and (6) difficulties in vision that were not corrected by the use of glasses or contact lenses. Exclusion criteria for the control group participants were the same as for those in the patient group.

The control group consisted of 38 healthy participants who met the same inclusion and exclusion criteria as the patient group participants, except for the diagnosis of depression. The three groups were matched by age, sex, and years of education (see Table 1).

**Table 1:** Demographic and clinical data of patients and control group.

**Items**	**Depressed without FSS (*n* = 30)**	**Depressed with FSS (*n* = 38)**	**Control (*n* = 38)**	**F/t/*x*^**2**^**	***P*-value**
Age (mean ± SD)	14.50 ± 1.51	14.72 ± 1.41	14 ± 1.7	0.29	0.77
Gender (male/female)	43.33%/56.67%	44.73%/55.27%	47.36%/52.64%	0.18	0.964
Education level (mean ± SD)	9.81 ± 0.74	9.84 ± 0.73	9.88 ± 0.58	−0.44	0.971
HDRS scores (mean ± SD)	15.66 ± 7.54	19.81 ± 6.95	/	−2.287	0.526
HAMA scores (mean ± SD)	13.43 ± 5.71	15.81 ± 5.97		−1.35	0.179
CSI scores (mean ± SD)	12.85 ± 11.46	72.86 ± 25.33	/	−12.824	0.00
Course of the disease (mean ± SD)	9.63 ± 6.77	9.76 ± 7.52		0.74	0.941
Episodes (first/recurrence)	22/8	30/8		4.35	0.113
Antidepressant (SSRIs/SNRIs)	28/2	33/5		0.79	0.452

*HDRS, Hamilton Rating Scale for Depression; HAMA, Hamilton Rating Scale for Anxiety; CSI, Children's Somatization Inventory; FSS, functional somatization symptoms; SSRIs, selective serotonin reuptake inhibitors; SNRIs, serotonin–norepinephrine reuptake inhibitors*.

### Clinical Symptom Assessment

In the patient group, depressive severity was assessed using the Hamilton Depression Rating Scale (HDRS) (34), while the degree of children's somatization symptoms was evaluated using the Children's Somatization Inventory (CSI), which was translated into Chinese from the revised English version of Garber (35).

The patient group was further divided into two subgroups based on participants' CSI scores, which measured the FSS in children and adolescents. The CSI has 49 items, divided into four factors: gastrointestinal symptoms, pain/weakness symptoms, cardiovascular and other symptoms, and pseudo-neurological symptoms. A 5-point Likert scale (0 = no, 1 = light, 2 = medium, 3 = lay particular stress on, and 4 = seriously) was used to respond to the items for each child's situation in the last 2 weeks, the highest total score on the CSI is 196 points. A score of 19 points and higher indicates the presence of FSS, and higher scores indicate higher severity of FSS. The Cronbach's alpha coefficient was 0.92 for the CSI, in this study, adolescents with scores of 19 or higher were assigned to the FSS group (*n* = 30), while the remaining children were assigned to the non-FSS group (*n* = 38).

### Emotional Memory Test

**Figure 1:**
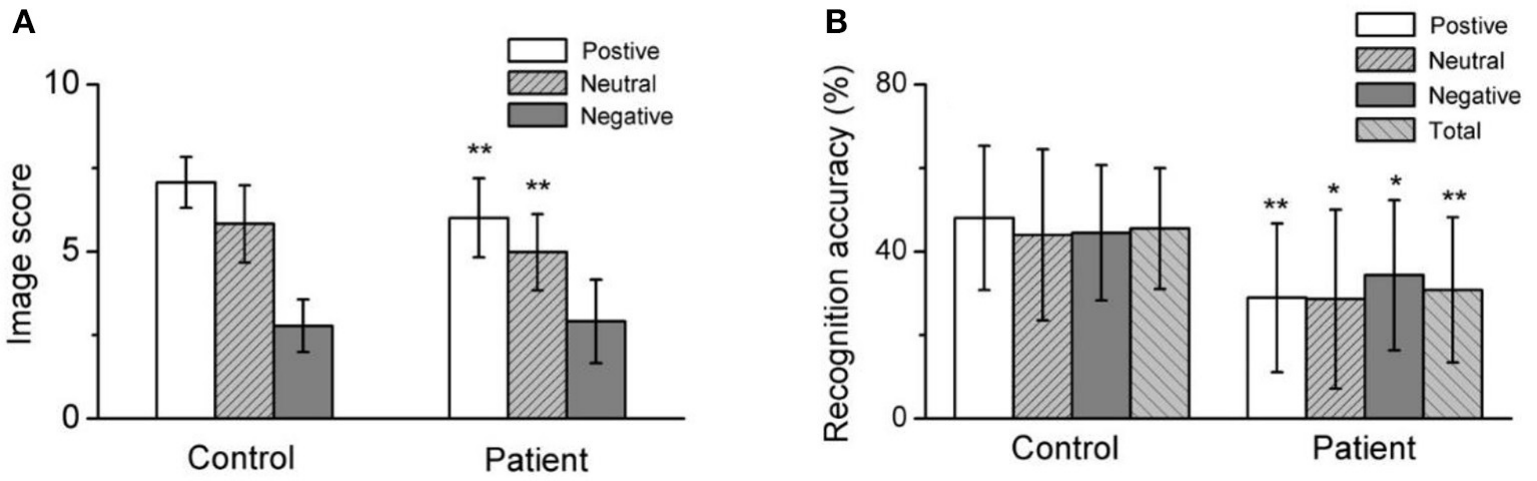
Both the positive image score and recognition accuracy, neutral image score and recognition accuracy, negative image recognition accuracy, and total recognition accuracy in depressed patients were significantly lower than healthy control. **(A)** Summarized data for the image score of different emotional image. **(B)** Averaged data for the recognition accuracy of different emotional image. *p < 0.05, **p < 0.001.

The emotional memory test paradigm has been used in clinical settings (36). Emotional memory was tested in two phases: unintentional learning and recognition. Pictures selected from the Chinese Affective Picture System (CAPS) were used as the emotional stimuli (37). A total of 90 photographs were selected; with, 45 being were chosen for the unintentional learning phase (15 photographs depicting emotionally neutral scenes, 15 depicting emotionally positive scenes, and 15 depicting emotionally negative scenes) and the other half being used as distractors during the recognition phase. During the first phase, each picture was presented for 3 s with an interval (no time limit) during which participants were required to rate the picture's emotional valence using a score between 1 (most negative) and 9 (most positive). The recognition phase was conducted 72 h after the first phase. In this phase, the two groups were presented with randomly mixed images on their computer screens. Participants were required to use the keyboard to indicate whether or no they had seen the picture previously. The experimental design is presented in Figure 1.

Each participant completed the task independently in the psychometric room, accompanied and supervised only by the tester. Both the first test and the retest were completed in the morning. The trials were conducted on a PC using the E-Prime software. We recorded the participant's valence rating for each picture and calculated the mean for each valence category (negative, neutral, and positive). We evaluated the participants' memory by measuring recognition accuracy (38), which was calculated as the difference between the hit rate and false alarm rate. The number of hits or false alarms was the number of old or distractor pictures identified as seen before by the participant. Hit rates and false alarm rates were calculated by valence category for each participant by dividing by the total number of hits and false alarms.

### Ethics Statement

This study involving human participants was reviewed and approved by the Medical Ethics Committee of the Hefei Fourth People's Hospital (number: HSY-IRB-PJ-AYXJJ-ZH001). Informed consent was obtained from all participants in this study, the consent form was signed by the participants themselves or their legal guardians.

### Statistical Analysis

Data were analyzed using SPSS (Version 22.0) in this study. The chi-square test and one-way analysis of variance (ANOVA) were used to analyze differences in participants' demographic characteristics. Differences in the clinical symptoms of depression were tested using the independent *t*-test. A one-way ANOVA and independent *t*-test were used to compare the differences in valence rating and recognition accuracy by valence (positive, neutral, and negative) between patient and control group participants. The Bonferroni correction *post-hoc* test and a covariance test were applied for comparing the two patient subgroups (with and without FSS). Pearson correlation analyses were performed to assess the relationship of the HDRS and CSI scores, respectively, with recognition memory performance, in the patient- group. A linear logistic regression model was used to investigate the association between recognition accuracy and the scores of HDRS and CSI scores, respectively. The significance level was set at *P* < 0.05 for a two-tailed test.

## Results

### Participant Characteristics

The demographic and clinical characteristics for the three groups (patients with FSS, patients without FSS, and control) are presented in Table 1. Mean normative control ratings for emotional valence and emotional arousal were calculated for set 1: positive photographs valence (M = 6.98, SD = 0.5) and arousal (M = 5.23, SD = 0.82), neutral photographs valence (M = 5.39, SD = 0.54) and arousal (M = 3.57, SD = 0.89), and negative photographs valence (M = 2.73, SD = 0.95) and arousal (M = 5.1, SD = 1.31). Mean normative control ratings for emotional valence and arousal for set 2 were also calculated: positive photographs valence (M = 6.70, SD = 0.77) and arousal (M = 5.48, SD = 0.71), neutral photographs valence (M = 5.58, SD = 0.61) and arousal (M = 3.32, SD = 1.20), and negative photographs valence (M = 2.74, SD = 0.97) and arousal (M = 4.97, SD = 1.08). There were no differences between the participants of the three groups in terms of age, gender, and years of education. The mean CSI score was significantly lower among patients without FSS than those with FSS in the patient group (12.85 ± 11.46 vs. 72.86 ± 25.33, *p* < 0.05). However, no such difference among the groups was observed in the mean HDRS scores (see Table 1).

### Valence Ratings and Recognition Memory

#### Adolescent Patient Group and Control Group

A comparison of the valence ratings for emotional images revealed that the ratings for positive and neutral images were significantly lower in the patient group (*t* = −4.942) than in the control group (*t* = −3.698, *P* < 0.001); however, there were no significant differences for the negative images (see Table 2).

**Table 2:** Comparative analysis of emotional memory between patient and control group ($\bar{X}$ ± *S*).

**Items**	**Patient *N* = 68**	**Control*N* = 30**	***t***	***P*^**a**^-value**
Positive image score	6.01 ± 1.18	7.07 ± 0.76	−4.942	0.000
Neutral image score	4.98 ± 1.14	5.83 ± 1.16	−3.698	0.000
Negative image score	2.91 ± 1.25	2.78 ± 0.79	0.655	0.5148
Positive recognition accuracy (%)	28.91 ± 17.82	48.05 ± 17.29	−5.317	0.000
Neutral recognition accuracy (%)	28.63 ± 21.45	44.00 ± 20.46	−3.568	0.001
Negative recognition accuracy (%)	34.32 ± 17.98	44.50 ± 16.21	−2.871	0.005
Total recognition accuracy (%)	30.80 ± 17.41	45.52 ± 14.49	−4.339	0.000
*F*	2.72	0.567		
*P*^b^-value	0.068	0.569		

*P^a^, P value for the comparison of recognition accuracy of emotional images between the two groups;*

*P^b^, P-value for the comparison of recognition accuracy of emotional images within each group*.

**Figure 2:**
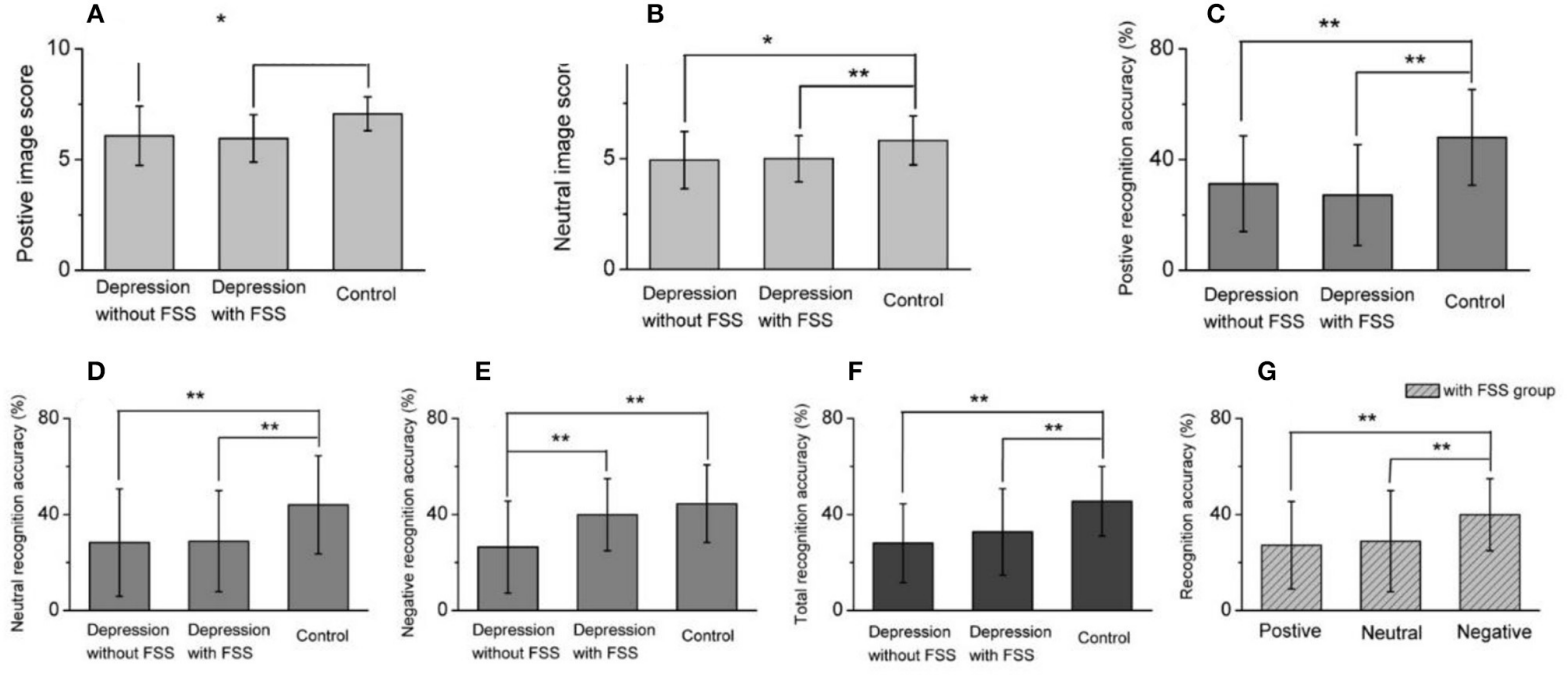
Comparison for image score or recognition accuracy between three groups and different image recognition accuracy within FSS group. **(A,B)** Summarized data for positive **(A)** and neutral **(B)** image score recognition accuracy, showing that both positive and neutral image score in depression with or without FSS were significantly lower than healthy control. **(C–E)** Comparative analysis for the averaged data of positive, neutral and negative image recognition accuracy, suggesting that the recognition accuracy of positive and neutral images in depression with FSS or without FSS was significantly lower than that of healthy control **(C,D)**, but in negative image recognition accuracy, depression without FSS was significantly lower than with FSS, synchronously lower than healthy control **(E)**. **(F)** Summarized data for the total recognition accuracy in three groups. **(G)** Within depression with FSS group, negative image recognition accuracy was significantly higher than positive or neutral image, respectively. *p < 0.05, **p < 0.001.

Furthermore, the recognition test scores revealed a significantly lower rate of recognition accuracy for positive (*t* = −5.317, *P* < 0.001), neutral (*t* = −3.568, *P* < 0.001), negative (*t* = −2.871, *P* < 0.001), and all (*t* = −4.339, *P* < 0.001) emotional images in the patient group than in the control group. However, comparative analysis suggested that there were no significant differences in the recognition accuracy rate of emotion images between the patient subgroups or between the patient and control groups (see Table 2 and Figure 2).

#### Differences Between Patient Group With or Without FSS and Control Group

A comparison of the scores by emotional images was conducted among the three groups. The results revealed that the positive and neutral image scores of patient groups with and without FSS were significantly lower than those of the control group (*F* = 12.208, *P* < 0.001; *F* = 6.801, *P* < 0.05, respectively). No such significant differences were found for the negative images, as shown in Table 3.

**Table 3:** Comparative analysis of emotional memory by group ($\bar{X}$ ± *S*).

**Items**	**Depression without FSS *N* = 38**	**Depression with FSS*N* = 30**	**Control *N* = 38**	***F***	***P*^**a**^-value**
Positive image score	6.08 ± 1.34	5.96 ± 1.07	7.07 ± 0.764	12.208	0.000
Neutral image score	4.94 ± 1.29	5.00 ± 1.04	5.83 ± 1.11	6.801	0.002
Negative image score	2.90 ± 1.21	2.92 ± 1.29	2.78 ± 0.79	0.173	0.842
Positive recognition accuracy (%)	31.30 ± 17.30	27.21 ± 18.21	48.05 ± 17.29	14.536	0.000
Neutral recognition accuracy (%)	28.33 ± 22.37	28.84 ± 21.07	44.00 ± 20.46	6.306	0.003
Negative recognition accuracy (%)	26.44 ± 19.13	39.92 ± 14.99	44.50 ± 16.21	9.907	0.000
Total recognition accuracy (%)	28.07 ± 16.40	32.74 ± 18.05	45.52 ± 14.49	10.316	0.000
*F*	0.415	5.446	0.567		
*P*^b^-value	0.662	0.006	0.569		

*P^a^, P-value for the comparison of recognition accuracy of emotional images between the three groups;*

*P^b^, P-value for the comparison of recognition accuracy of emotional images within each group*.

The results also revealed that the patient group with and without FSS had a significantly lower recognition accuracy rate for positive and neutral emotion images and total recognition accuracy rate than the control group (*F* = 14.536, *P* < 0.001; *F* = 6.306, *P* < 0.05; *F* = 10.316, *P* < 0.001, respectively). On the other hand, the recognition accuracy rate for negative emotion images was significantly lower in the patient group without FSS than in the patient group with FSS and the control group (*F* = 9.907, *P* < 0.001).

**Figure 3:**
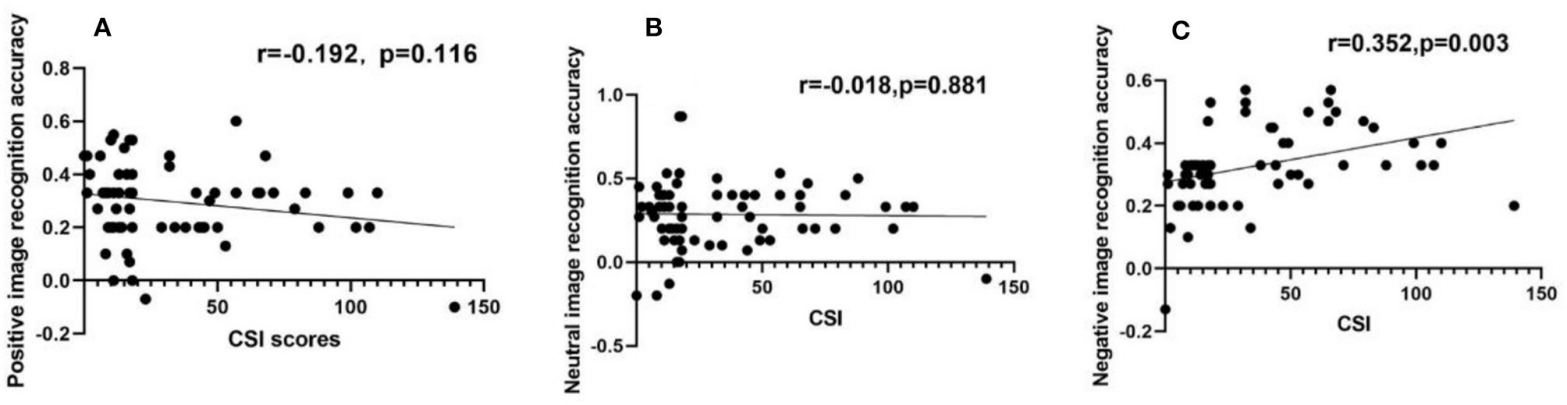
Negative image recognition accuracy had a positive correlation with CSI scores, while positive or neutral image recognition accuracy had no relation with CSI scores, respectively. **(A–C)** Scatter diagram for the recognition accuracy of positive image **(A)**, neutral image **(B)**, and negative image **(C)**, showing that positive or neutral image recognition accuracy had no relation with CSI score **(A,B)**, but there was a positive correlation between negative image recognition accuracy and CSI score **(C)**.

Comparative analysis within the patient subgroups suggested that the recognition accuracy rate of negative emotion images was significantly higher than that of positive and neutral emotional images in the subgroup with FSS (*F* = 5.446, *P* < 0.05). The covariance analysis between the patient subgroups after controlling for HDRS scores showed that the recognition accuracy rate of negative images in the subgroup without FSS was significantly lower than in the FSS subgroup (*F* = 17.71, *P* < 0.001) (see Table 3 and Figure 3).

### Correlation Analyses

**Figure 4:**
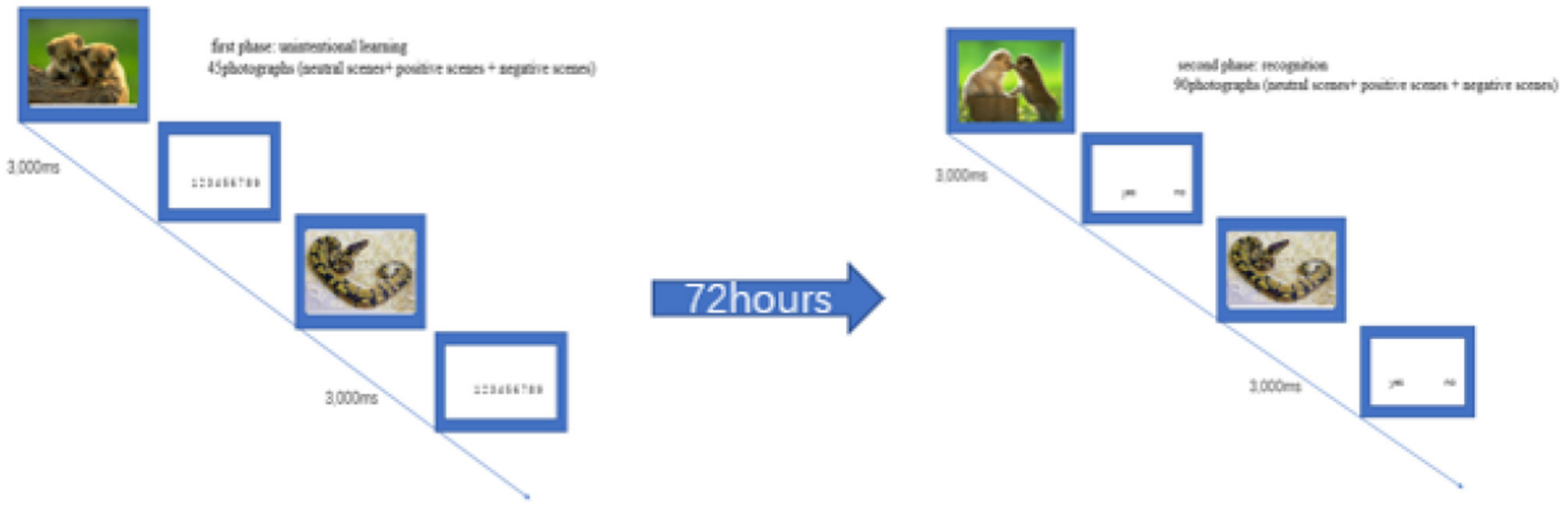
Examples of one positive and negative target picture in two phases of emotional memory test (unintentional learning and recognition).

SCI scores for the patient group had a positive correlation with the recognition accuracy rate of negative emotion images (*r* = 0.352, *P* < 0.05), as shown in Table 4 and Figure 4. However, no other significant correlations were found with HDRS scores.

**Table 4:** Correlated analysis between emotional images with HDRS and CSI scores (*n* = 68).

**Items**	**Positive image recognition accuracy**	**Neutral image recognition accuracy**	**Negative image recognition accuracy**
HDRS score R value	−0.097	−0.079	−0.243
*P*-value	0.465	0.530	0.510
CSI score R value	−0.192	−0.018	0.352
*P*-value	0.116	0.881	0.003

Additionally, the linear regression models revealed that the CSI scores predicted the recognition accuracy rate of negative and positive emotion images (b = 0.447, *P* < 0.05; b = −0.299, *P* < 0.05, respectively). Moreover, it was observed that an increase in CSI scores led to an increase and decrease in the recognition accuracy rate of negative and positive emotion images, respectively, as shown in Table 5.

**Table 5:** Predictors generated by multiple linear Regression with CSI as dependent variables (*n* = 68).

**Variables**	**Unstandardized coefficients**			**Standardized coefficients**	***p*-value**	**95.0% confidence interval for B**	
	**B**	**Std. Error**	***T***			**Lower bound**	**Upper bound**
Positive image recognition accuracy	−63.472	27.677	−2.293	−0.299	0.025	−118.764	−8.181
Neutral image recognition accuracy	−4.964	21.712	−0.229	−0.031	0.820	−48.339	38.441
Negative image recognition accuracy	111.417	29.996	3.714	0.447	0.000	51.493	171.314

## Discussion

To the best of our knowledge, this is the first study to examine the emotional memory of Chinese adolescent patients with depression, with or without FSS. The key findings can be summarized as follows: First, the positive and neutral image ratings of adolescent patients with depression were significantly lower than those of the control group participants, regardless of FSS. Second, adolescent patients with depression reported significantly lower recognition accuracy rates than control group participants for all three types of images; however, there were no differences in recognition rates of the three types of images among patients with depression. Thus, adolescent patients with depression had no obvious bias for negative emotional memory. Third, while both patient subgroups showed significantly lower recognition accuracy than the control group for positive and neutral images, the patient group without FSS showed significantly lower recognition accuracy than both the patient group with FSS and the control group for negative images; this difference persisted even after controlling for HDRS scores. Lastly, the recognition accuracy rate for negative emotion images was significantly higher than that of positive emotion image and neutral emotion image in patients with FSS, suggesting that depression patients with FSS had a bias for negative emotional memory. Furthermore, the recognition accuracy hit rate for negative images was positively correlated with the CSI score, but no such correlation was found for the recognition accuracy hit rate for positive and neutral images in the patient group.

This study suggest that adolescent patients with depression have a lower degree of arousal in response to positive and neutral stimuli, regardless of FSS. The existing literature indicates that healthy participants' emotional response to positive stimulation is stronger than that of those with first episode depression, suggesting that healthy people have the characteristic of positive emotional bias (39). In addition, various cognitive biases have been reported in patients with depression, such as perceptual bias (negative emotion potency), attention bias, and interpreactive bias (40, 41).

Patients with depression show a decrease in pleasure and arousal from positive vocabulary and an increase from negative vocabulary (42). Patients with somatoform disorders are sensitized to the perception of symptoms, with the attention and exaggeration of sensory signals, they are more likely to experience somatoform symptoms (43, 44). The amygdala receives and integrates all incoming information from the sensory system, cognitively assesses this sensory information, identifies the threats or dangerous cues, generates negative emotions, and attaches meaning to all the information received, especially the negative experiences (45). Therefore, people with depression and somatization symptoms are more likely to be aroused by negative images. Furthermore, results suggest that arousal and valence may operate using distinct neural pathways to mediate the effect of emotion enhancement on memory formation (46, 47). In adolescent patients with depression, the stimulating ability of positive and neutral emotion images decreased, but the stimulation of negative emotion images was unaffected. This finding indicates that the arousal mechanisms may vary for different types of emotion images.

Our study found that adolescent patients with depression have emotional memory impairment but no obvious bias for negative emotional memory was found. In one previous study, the recognition accuracy rate for positive emotion images was significantly lower among adult patients of depression than among healthy controls. Although, there was no statistical difference in the recognition accuracy rate for neutral and negative emotion images between adult patients and healthy controls, the recognition accuracy rate for negative emotion images was significantly higher than that of positive and neutral images among patients with depression. These findings indicate that there is a negative emotional bias in adults with depressive disorders (5). In another study, adult patients with depression showed lower overall memory scores on recall tests and tended to remember negative words better, and the severity of depression was also related to the remembrance of fewer positive words and more negative words (42). In a previous study of working memory, the recognition accuracy rate in adult patients with depression was significantly lower than that in healthy participants, and the reaction time for the former group was significantly prolonged (48).

Additionally, studies on the working memory of adolescents with depressive disorders confirm that they do not give priority to angry faces, that is, negative memory biases (49). The negative memory biases of patients with depression may be mainly caused by inhibitory dysfunction (50). Emotional memory inhibition research has found that, compared to neutral stimuli, negative stimuli improved the recognition accuracy rate of both recall and inhibitory recall conditions, indicating that negative stimuli are not only easier to remember, but also more difficult to be forgotten (51). However, a study that utilized the emotional Stroop task for children with depression aged 13 to 15 years showed no significant difference in response inhibition function between the group with depression and the healthy control group (52). Thus, it can be seen that the neurophysiological activities related to emotional memory in adolescent patients with depressive disorder are different from those in adult patients with depressive disorder.

Our research findings suggest that the positive and neutral emotional memory is impaired and negative emotional memory is retained in adolescent patients of depression with FSS. Furthermore, our findings imply that adolescent patients with depression and FSS have preferences for negative memory, and somatic symptoms may be related to negative memory retention in these patients. Studies of somatization disorder have found that participants with somatoform symptoms showed a memory bias for illness-related stimuli in the word-stem completion task. Moreover, this effect could not be explained by comorbid depression (32) and may be due to different pathological mechanisms of emotional memory between adolescent patients of depression with and without somatic symptoms. Studies show that when somatic symptoms are experienced, the parallel activation of human perception and memory systems produces many perceptual hypotheses aligned with the experience. The primary attentional system (PAS) in patients with FSS selectively pays attention to the “abnormal representation” of somatic symptom information. Due to defects in the process of automatic activation of perception by PAS, the secondary attentional system (SAS) pays selective attention to information such as body sensations, disease information, and negative effects, and the expected physical discomfort interacts with the patient's memory (24). Thus, people with depression and somatic symptoms assign more attention to negative stimuli, thereby, producing stronger negative memories.

The amygdala—the most important brain structure for emotional memory and response—facilitates emotional evaluation, is at the core of the entire emotional memory neural network and plays an important role in activating, consolidating, and processing emotional memory (53). Emotional enhancement effect of memory (EEM) shows that the recall rate of emotional information is faster and more accurate than neutral information; furthermore, increasing the degree of emotional information can enhance memory and promote emotional memory encoding, consolidation, and retrieval when the basolateral amygdala and hippocampus structures are activated (54). Negative stimulation can enhance memory ability in comparison to neutral stimulation; this phenomenon may be associated with the role of emotional events (especially negative stimulation) in improving the excitability of amygdala neurons and their neural pathway activities between the hippocampus and cerebral cortex; thus, strengthening the consolidation of declarative memory. The effect of negative emotion on memory enhancement may depend on the degree of activation and emotional arousal of the amygdala and the degree of interaction between the amygdala and hippocampus (55). The loss of emotional memory is specifically related to the connectivity of the medial prefrontal cortex and hippocampus (56). Studies using MRIs have shown that the resting state functional connectivity (RAFC) in the amygdala, basal dorsolateral prefrontal cortex (DLPEC), and ventromedial prefrontal cortex (VMPFC) is decreased for adolescents with depression, and the function of the frontal edge circuit in emotional regulation is discontinued (57). This neural mechanism may cause emotional memory impairment among adolescent patients with depression.

Moreover, somatic symptoms and emotional disorders may have a common neurophysiological mechanism. Existing studies have shown dysfunctions in certain brain regions of patients with somatization disorder, such as the amygdala, frontal lobe, anterior cingulate, and specific limbic cortex; this damage is associated with pain and mood regulatory system circuits (58–60). Specifically, patients with somatization disorder showed overactivation of VM PFC, fusiform gyrus, and insular lobe in response to negative emotion-regulated pain stimuli (61). When nociceptive stimulation was provided without emotional background, patients with somatization disorder reported overactivation of the amygdala, insular lobe, somatosensory cortex, and inferior parietal cortex, and lower activation of the ventromedial prefrontal/preorbital cortex (62). Therefore, the evidence suggests differences in the neural pathways of patients with depression with and without FSS. These differences may exist in the amygdala, and affect emotional memory function. However, its neural mechanisms need to be studied further.

There are several other limitations to this study. First, the small sample size for patients of depression with and without FSS affected the reliability of the results. Second, the effects of medication on the memory of emotional stimuli remain unclear. Several participants were on antidepressants in the current study. Lastly, the current study is limited due to the cross-sectional nature of its design.

In conclusion, this study found that adolescent patients with depression, with or without FSS, have different degrees of emotional memory impairment. Negative emotional memory impairment was not found in patients of depression with FSS, and it was related to the CSI scores. Somatic symptoms may be a factor affecting emotional memory in adolescents with depressive disorders. These results will provide a clinical basis for planning treatment and interventions for adolescent depression.

## Data Availability Statement

The raw data supporting the conclusions of this article will be made available by the authors, without undue reservation.

## Ethics Statement

The studies involving human participants were reviewed and approved by Medical Ethics Committee of the Hefei Fourth People's Hospital (number: HSY-IRB-PJ-AYXJJ-ZH001). Written informed consent to participate in this study was provided by the participants' legal guardian/next of kin.

## Author Contributions

MD had full access to all the data in the study and took responsibility for the integrity of the data and the accuracy of the data analysis and drafting of the manuscript. ZH, MD, and CX: concept and design. MD, LX, LS, HS, and CB: acquisition, analysis, or interpretation of data. MD and GP: statistical analysis. ZH obtained funding and supervision. All authors critical revision of the manuscript for important intellectual content.

## Conflict of Interest

The authors declare that the research was conducted in the absence of any commercial or financial relationships that could be construed as a potential conflict of interest.

## Publisher's Note

All claims expressed in this article are solely those of the authors and do not necessarily represent those of their affiliated organizations, or those of the publisher, the editors and the reviewers. Any product that may be evaluated in this article, or claim that may be made by its manufacturer, is not guaranteed or endorsed by the publisher.
